# Tumor Embolic Stroke: The Importance of Pathological Assessment of Clots after Thrombectomy

**DOI:** 10.3390/jcm13071834

**Published:** 2024-03-22

**Authors:** Richard Baker, Zohabe Bakali, Jeffrey S. Crocker, Ashkan Mowla, Matthew Smith, Aaron Grossman, Matthew C. Hagen, Charles J. Prestigiacomo, Peyman Shirani

**Affiliations:** 1 College of Medicine, University of Cincinnati, Cincinnati, OH 45267, USAhagenmw@ucmail.uc.edu (M.C.H.);; 2 Department of Pharmacology & Systems Physiology, College of Medicine, University of Cincinnati, Cincinnati, OH 45267, USA; 3 Department of Neurosurgery, University of Southern California, Los Angeles, CA 90033, USA; mowla@usc.edu; 4 Department of Neurology & Rehabilitation Medicine, College of Medicine, University of Cincinnati, Cincinnati, OH 45267, USA; 5 Department of Neurosurgery, College of Medicine, University of Cincinnati, Cincinnati, OH 45267, USA; 6 Department of Pathology & Laboratory Medicine, College of Medicine, University of Cincinnati, Cincinnati, OH 45221, USA

**Keywords:** tumor, stroke, embolism, pathology, thrombectomy, ESUS, CVD, malignancy, cancer

## Abstract

While tumor emboli are a rare cause of stroke in cancer patients, they highlight the importance of gross observations and pathological assessments in the evaluation of clots. In this case report, a 70-year-old male with type 2 diabetes mellitus and coronary artery disease presented with acute left-sided weakness. He was clinically diagnosed with stroke and given alteplase at 1.5 h from last known normal. He then underwent CT angiography that showed right internal carotid artery occlusion and immediate thrombectomy. The recovered clot was white and lipid-like; due to its atypical appearance, it was sent for pathological assessment, where it was shown to bear features of malignancy. Subsequent imaging identified masses indicating malignancy in the left gluteus, right pleural hilum, and spine. Tumor embolic stroke is a rare pathology. Embolic diseases such as strokes and pulmonary embolisms are common in patients with cancer. Embolic stroke of undetermined source (ESUS) represents a significant portion of cancer strokes. Tumor emboli, though rare, may be an underappreciated source of ESUS in cancer patients. We intend for this case to demonstrate the value of pathological assessment for atypical thrombi as well as highlight the etiology of tumor embolic strokes.

## 1. Introduction

While tumor emboli are a rare cause of stroke, with few reported cases that are most frequently assessed post-mortem [1,2,3,4,5,6,7,8,9,10,11,12], they represent an etiology of stroke for which traditional management would be insufficient. When tumor embolic strokes occur, they are often the result of tumors that are present in or metastasize to the heart [13,14], and may affect other tissues such as the limbs [3,4,5,6,7,8,9,10,11,12,13,15,16]. Roughly half of strokes in cancer patients are embolic strokes of undetermined source (ESUS) [17]. These strokes can represent any etiology not identified by typical assessment, and may include multiple additional etiologies, including tumor emboli [17]. Multiple other brain injuries such as mass effects, tumor hemorrhages, or arterial/venous invasions may also create acute strokes or stroke-like events in patients with cancer [18]. Due to the high potential for non-standard causes of stroke in patients with cancer, identification of atypical thrombi and pathological assessment may prove to be a useful boon to care, as well as help to better characterize unknown etiologies of stroke. This case aims to emphasize the importance of pathological assessment of atypical thrombi and the features of rare tumor emboli.

## 2. Case

**Figure 1:**
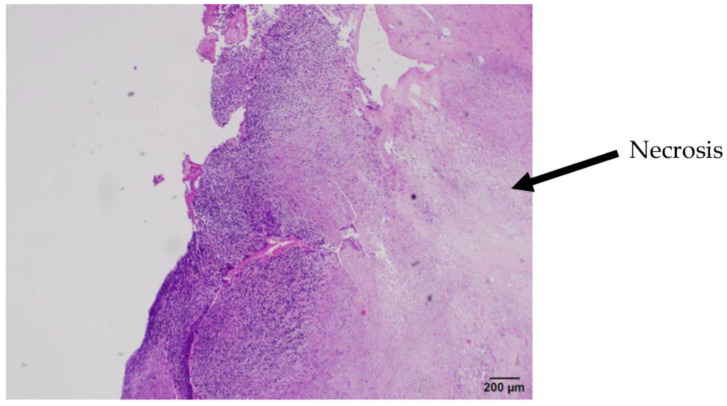
Microscopic image of tumor with HE staining. Image shows necrosis (right) consistent with malignancy.

**Figure 2:**
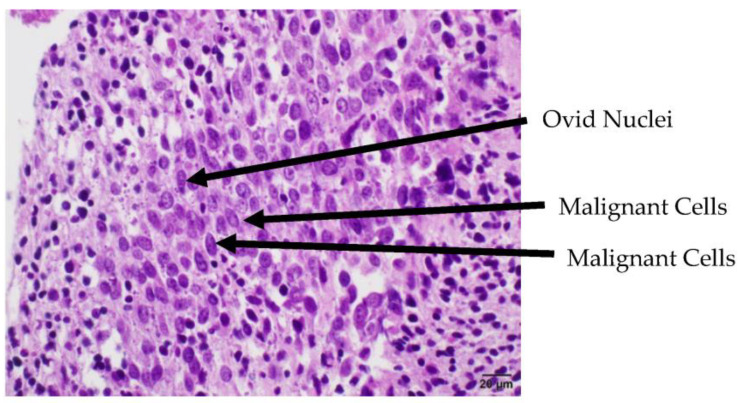
High-power microscopic image of tumor with HE staining. Image shows ovid nuclei and cells with scant cytoplasm, suggesting malignancy.

**Figure 3:**
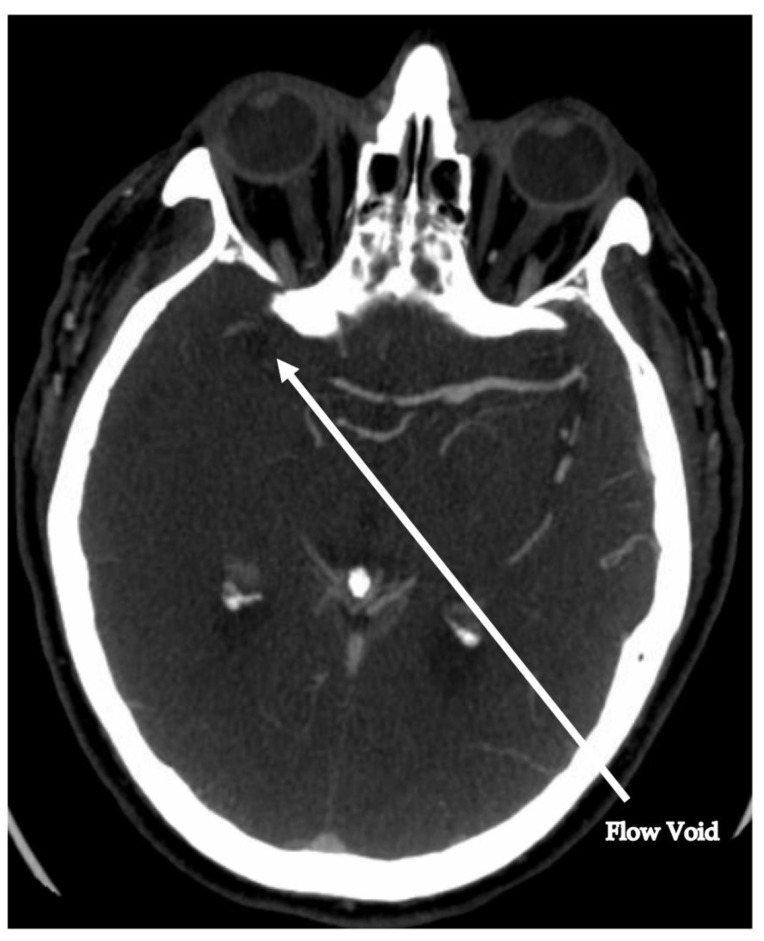
CT angiogram showing flow void in the right internal carotid artery.

In January 2018, a 70-year-old male with history of hypertension, type 2 diabetes mellitus, and coronary artery disease presented to the Kettering hospital emergency room after acute onset of left-sided hemiparesis and anesthesia including left-extremity limp and facial droop. Roughly one week prior to presentation, he had a left hip hematoma drained, during which a high-grade sarcoma was revealed. Further characterization of the sarcoma or assessment for metastasis had not been performed at time of admission. He was clinically diagnosed with a right-sided ischemic stroke (NIHSS 17). EKG showed no arrhythmia. Head non-contrast CT revealed no intracranial bleeding, and he was transferred to the University of Cincinnati Medical Center after receiving thrombolysis at 1.5 h after his last known normal. Subsequent CT angiography of the head and neck showed distal right internal carotid artery occlusion. He underwent a thrombectomy via right femoral artery access, resulting in TICI 2B reperfusion after removal of a clot from the right ICA. He received standard care post thrombectomy (Figure 1, Figure 2 and Figure 3).

**Figure 4:**
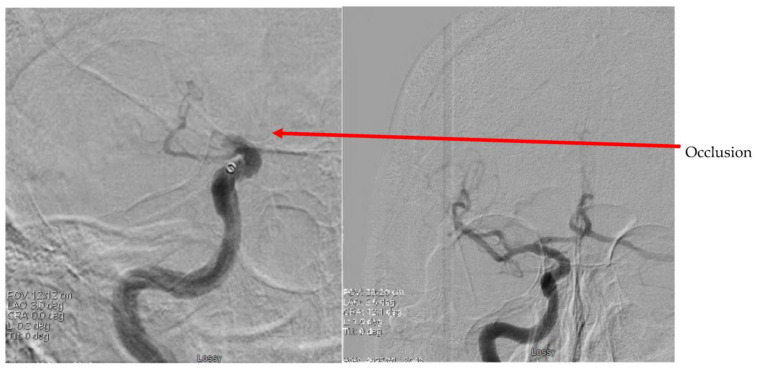
Pre-thrombectomy (**left**) and post-thrombectomy (**right**) subtraction diagnostic cerebral angiography showing occlusion and reperfusion of the right internal carotid artery and its branches.

**Figure 5:**
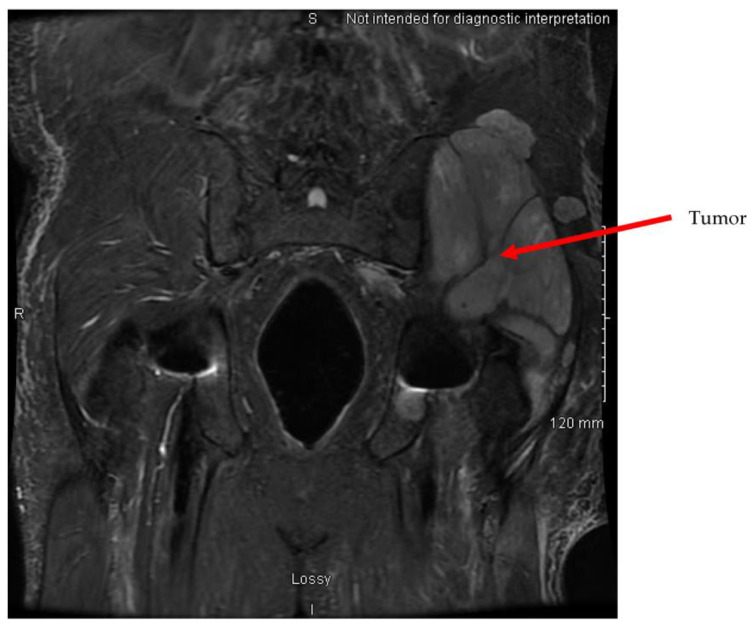
MRI abdomen pelvis, extensive tumor in left hip appears bright on T2 coronal MRI.

**Figure 6:**
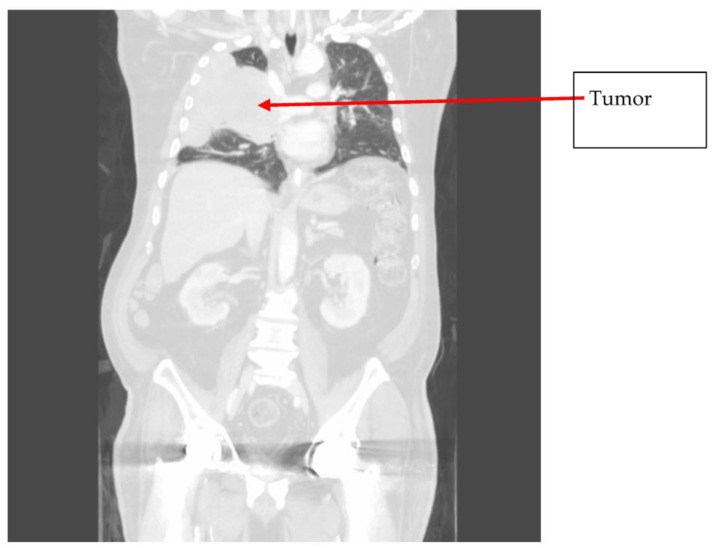
Chest CT showing extensive tumor of the right upper lobe.

Gross assessment of the clot demonstrated an atypical white, fatty appearance. Due to the atypical features of the clot inconsistent with a typical thromboembolism, it was sent for pathological assessment. Pathological assessment of the thrombus showed it consisted of monomorphic cellular proliferation composed of spindle-shaped cells with ovid and irregular hyperchromatic nuclei present in sheets. Foci of apoptosis and prominent necrosis in the central portion of the tissue were noted. These findings were suggestive of malignancy, indicating that the embolus was a tumor fragment rather than a thromboembolism. Immunohistochemical stains for CD 20, CD3, CD68, and CD45 were negative in the cells of interest, which was not supportive of a possible hematological neoplasm. Subsequent imaging showed central necrotic masses in the left gluteal musculature indicative of malignancy. Lytic spinal lesions in the thoracic and lumbar spine were also identified. CT of the chest identified a large, heterogenous, multilobulated mass in the right pleura and hilum consistent with malignancy. The mass occluded the right upper and middle lobe airways and encased the right pulmonary arteries. Radiology assessment indicated the lung mass was likely the primary tumor; however, the site of tumor origination was not identified prior to the patient entering hospice care. Metastasis to the heart was not observed (Figure 4, Figure 5 and Figure 6).

Post-thrombectomy MRI of the head without contrast identified injury to the right temporal, frontal, and parietal lobes as well as injury to the basal ganglia and right caudate nucleus. No findings consistent with head, neck, or brain metastases were identified.

The patient’s family requested the patient receive hospice care. Consequently, no further evaluation of the tumor’s origin was completed. At discharge, the patient demonstrated hemiparesis of the left upper and left lower extremities. 

## 3. Discussion

This case describes an unusual identification of a tumor embolic stroke. Tumor embolic stroke is a rare but identified phenomenon. Most existing case reports describe post-mortem identification of tumor embolic stroke, and most of these tumor emboli causing stroke or peripheral artery embolization originate in the heart, not the pulmonary vasculature [1,2,3,5,6,7,8,9,10,11,12,13,18,19].

Ischemic vascular disease is one of the common complications of cancer. Strokes and pulmonary emboli can be an early presentation of cancer. Cerebrovascular disease is estimated to occur in ~15% of patients with non-central nervous system cancers [20], with a significant increase in stroke risk observed in lung, breast, pancreatic, and colorectal cancers [21]. The increased incidence of thromboembolism in cancer may be attributed to some specific pathologies tied to malignancy. Patients with cancer who experience stroke have been observed to have increased expression of atypical genes associated with hypoxia and inflammation, as well as increased concentration of prothrombotic factors and microparticles in the bloodstream [22,23,24,25,26]. Direct tumor effects, such as arterial or venous invasion, blood vessel compression, intratumoral hemorrhages, and tumor emboli, have also been described [19].

Notably, in the rare instances where tumor embolisms have been identified, they occurred in cases such as cardiac liposarcoma, renal cancer with metastasis in the pulmonary vein and heart, and lung cancer [1,2,3,18,19]. Many of these cases occurred in patients with a prior diagnosis of cancer and metastasis to the heart with cardiac masses as the probable nidus of the tumor emboli [4,13,15]. Among cardiac tumors, left atrial and aortic valve tumors have the greatest risk of tumor embolism [13], with left atrial myxomas carrying a 45% risk of tumor embolization [15]. In one case, the embolism was noted to have atypia, prompting an evaluation, and it was discovered that the embolism was in fact malignant in origin [4], suggesting such tumor emboli frequently appear atypical when extracted. These direct tumor effects are consequential because, unlike traditional thromboembolisms, they would not be susceptible to alteplase. Alteplase cleaves plasminogen into plasmin, which can break up the fibrin structure composing thromboembolisms; however, tumor emboli are largely not composed of fibrin and platelet products and thus are not susceptible to plasminogen [14].

Despite the multiple identified mechanisms of cancer-related stroke, these strokes are largely mysterious, and in many cases, the precise pathophysiology may be difficult to identify [17]. Roughly half of strokes occurring in cancer patients are classified as embolic stroke of an undetermined source (ESUS), meaning they are non-lacunar ischemic strokes without a clear etiology despite standard evaluation (echocardiography, CT angiography, EKG) [17]. This does not include cryptogenic strokes with incomplete evaluation [16,27,28], but may include strokes with non-traditional mechanisms not identified in the standard post-stroke work-up [16,17,27,28].

ESUS likely represent a heterogenous set of diseases; however, most are assumed to come from one of seven sources: “atrial cardiopathy, covert atrial fibrillation, left ventricular disease, atherosclerotic plaques, patent foramen ovale, cardiac valvular disease, and cancer” [28]. Stasis is presumed to be the primary generator of thrombus formation [28]. ESUS is generally assumed to be thromboembolic [28]; however, it is possible that many of these cryptogenic, mysterious embolisms represent alternative pathologies such as tumor embolism.

However, there is evidence that aspirin is non-inferior to direct oral anticoagulants in the management of ESUS [29], suggesting that the traditional embolic etiology may not be the source of strokes described as ESUS. Non-standard etiologies, such as tumor embolic stroke, cancer-mediated hypercoagulability, and vascular injury from chemotherapy or radiation, may also promote ESUS in cancer patients [17]. Indeed, these sorts of mechanisms would explain the apparent ineffectiveness of anticoagulation [29], which would not be effective for mechanisms from direct tumor involvement, though this effect is observed in non-cancer ESUS as well [29]. Therefore, tumor involvement is, at minimum, not fully explanatory. The aforementioned direct tumor involvement may also play a role in the development of ESUS in cancer patients if the mechanism cannot be clearly determined [18].

Navi et al. argue that ESUS associated with cancer may be better thought of as a separate category of disease in large part due to the absence of traditional risk factors (cancer patients who experienced ESUS have lower BMIs and fewer atherosclerotic factors than noncancer patients with cryptogenic strokes or cancer patients with known-mechanism strokes) [28,30], increased severity, increased risk of recurrence, and association with disseminated adenocarcinoma [17]. They are also associated with increased D-dimer and inflammatory markers, they are frequently present in bilateral anterior and posterior circulation, and they appear to be associated with higher rates of recurrence, early neurological injury, and early mortality [17]. Patients with ESUS associated with cancer also appear to have worse outcomes compared to patients with ESUS without cancer [17]. The hypothesized mechanisms of cancer-associated ESUS include nonbacterial endocarditis secondary to cancer or cancer therapy, paradoxical emboli, or tumor emboli [25]; however, a unifying etiology or a set of etiologies describing cancer-associated ESUS has been established [24]. The heterogenous nature of these etiologies means that effective prophylactic treatment may vary considerably between subtypes.

While tumor emboli are rarely observed [13,15], there is reason to suspect the diagnosis may be more common than reported, particularly given the large number of ESUS in cancer patients [17]. It is easy to imagine tumor vegetations that originate in or spread to the lungs breaking off into pulmonary veins [23]. Navi et al. reported that tumor emboli typically result from pulmonary and cardiac tumors through precisely this mechanism, though this claim is not cited [23]. Stergiopoulos et al. described an incidence of pulmonary tumor emboli secondary to metastatic renal cancer, noting that origination from pulmonary veins appeared to be a novel nidus for tumor embolisms [4]. Grazziotin et al. reported that pulmonary tumors are, in fact, the most common source of tumor emboli resulting in arterial embolization [4].

The involvement of pulmonary tumors would make sense. Given the lack of fixed vascular barriers (cardiac valves are temporary barriers) between the pulmonary veins and systemic circulation, pulmonary nidus would seemingly be a common source of tumor emboli. While the location of tumor embolism was not determined in this case, given the extensive pulmonary involvement, it appears likely that a pulmonary venous, rather than cardiac, tumor was the source of the embolism—indeed, no cardiac tumor was clearly identified on imaging. Many features of this case resemble the case described by Stergiopoulos et al., such as the suspected pulmonary origin of the tumor embolism and the injury to multiple brain regions [3]. A paradoxical tumor embolism could also be a possible explanation for this patient’s tumor embolism, but would appear less likely than a pulmonary origin.

Further, the fact that lung cancer carries a high stroke risk, and that disseminated adenocarcinoma is associated with ESUS, is consistent with the hypothesis that tumor emboli may break off from pulmonary cancers and cause strokes and that tumor emboli may be an explanation for some apparent ESUS in patients with cancer [21,24]. Indeed, most tumor emboli do occur in cancers that originate in the heart or lungs or metastasize to those locations [1,2,3,4,5,6,7,8,9,10,11,12]. It is important to remember none of these findings demonstrate that tumor embolisms explain even a consequential portion of ESUS in patients with cancer, and while all of the aforementioned observations are consistent with the notion that tumor embolism may be more common than supposed, they are not specific to tumor embolism.

Nevertheless, given the frequency of otherwise unaccounted for strokes in cancer patients [17], removal of clots and pathological assessments may be warranted as a general practice. Atypical clots may represent unusual etiologies such as tumor embolic stroke, and a lack of assessment may lead to an underappreciation of the true burden these sorts of etiologies cause.

Given the relatively common phenomenon of tumor pulmonary embolism, and the propensity of most cancers to spread to the lungs, it seems odd that tumor embolic stroke is not a more common phenomenon.

One explanation for this may be that tumors rarely form fragments large or stable enough to cause strokes. Tumor fragments may be too small to occlude vessels, or too loosely held together to withstand the sheer force of blood flow.

It is also possible that pulmonary tumors rarely serve as niduses of tumor emboli; many of the described cases show tumor emboli that are sourced to the cardiac tissue from either metastasis or native cardiac cancers [1,2,3,4,5,6,7,8,9,10,11,12]. While pulmonary metastasis is very common, cardiac metastasis is not as common and cardiac cancers occur less frequently.

Another explanation is that tumor emboli are, in fact, more common than supposed, but are likely to cause lacunar strokes. In this speculative scenario, vaso-invasive tumor fragments that break off from pulmonary or cardiac tumors may cause small and unnoticed—and therefore unretrieved—occlusions of subcortical vasculature [19]. These cancer patients may present with symptoms similar to vascular dementia, which may be difficult to distinguish from more obvious causes of cognitive decline such as fatigue, chemotherapy, or endocrine dysregulation. One finding that may be consistent with this would be an increased frequency of brain metastasis in cancer patients showing signs of vascular dementia compared to cancer patients without signs or symptoms of vascular dementia, as, unlike typical atherosclerotic or thromboembolic clots, tumor emboli are likely to still be living tissues which can metastasize into infarcted brain tissue. Indeed, Navi et al. suggest that survivors of tumor embolic strokes are more likely to have metastasis at the site of occlusion [17].

While it seems plausible that tumor embolism may be a more common etiology of ESUS in cancer patients than is currently assumed, the frequency of more traditional etiologies of stroke means that, in acute situations, standard protocols such as alteplase and thrombectomy would still be ideal when available. While alteplase would not be effective in dealing with tumor emboli, there is no reason to suspect it would cause specific harm in a patient otherwise suitable for alteplase beyond increased risk of bleeding, which would be acceptable when thromboembolism is still the most likely etiology of stroke.

What is demonstrated by the existence of tumor embolic stroke is the necessity of pathological assessment in patients with atypical clots, as the discovery of an atypical etiology may guide further care, as well as better represent the frequency of etiologies that may be undercounted. Given the frequency of ESUS in cancer patients [17], pathological evaluation of clots may be particularly helpful at identifying underappreciated etiologies.

## 4. Conclusions

This case demonstrates the value of pathological assessments of emboli with unusual characteristics. The atypical features of this thrombus revealed that it was a malignant mass rather than a more typical cholesterol embolus or thromboembolism. These sorts of tumor embolisms appear most commonly in patients with cardiac or pulmonary lesions [1,2,3,4,5,6,7,8,9,10,11,12], as was the case in this patient. Identification of this sort of embolic stroke alters post-stroke care and prevention strategies [29]; consequently, care would need to be adjusted to better reflect a rare etiology, primarily due to the detection of an atypical source of stroke. Given the frequency of atypical stroke etiologies in cancer patients [17], the assessment of removed clots, particularly atypical ones, is a useful step in immediate patient care and in the general characterization of strokes as a phenomenon.

## Data Availability

The original contributions presented in the study are included in the article, further inquiries can be directed to the corresponding author.
